# Patterns of Gray and White Matter Volume Alterations in Patients With Post-Traumatic Anosmia: A Voxel-Based Morphometry Study

**DOI:** 10.3389/fneur.2022.690760

**Published:** 2022-07-04

**Authors:** Xing Gao, Baihan Su, Zhifu Sun, Lei Xu, Yongxiang Wei, Dawei Wu

**Affiliations:** ^1^Department of Otorhinolaryngology-Head and Neck Surgery, Children's Hospital, Capital Institute of Pediatrics, Beijing, China; ^2^Department of Otolaryngology, Smell and Taste Center, Beijing Anzhen Hospital, Capital Medical University, Beijing, China; ^3^Department of Medical Imaging, Beijing Anzhen Hospital, Capital Medical University, Beijing, China; ^4^Department of Otorhinolaryngology-Head and Neck Surgery, Peking University Third Hospital, Beijing, China

**Keywords:** post-traumatic olfactory dysfunction, magnetic resonance image, olfactory cortex, gray and white matter volume, traumatic brain injury

## Abstract

**Objective:**

Traumatic brain injury is one of the major causes of human olfactory dysfunction and leads to brain structure alterations, mainly in the cortical olfactory regions. Our study aimed to investigate volume changes in the gray matter (GM) and white matter (WM) in patients with post-traumatic anosmia and then to explore the relationship between GM volume and olfactory function.

**Methods:**

Ethics committee approved prospective studies which included 22 patients with post-traumatic anosmia and 18 age- and gender-matched healthy volunteers. Olfactory function was assessed using the Sniffin' Sticks. High-resolution 3-dimensional T1 MRIs of the participants were acquired on a 3T scanner and the data were collected for voxel-based morphometry (VBM) analysis. Furthermore, the GM and WM volumes of the whole brain regions were compared and correlated with olfactory function.

**Results:**

The analysis revealed significant GM volume reduction in the orbitofrontal cortex (OFC), gyrus rectus (GR), olfactory cortex, insula, parahippocampal, temporal pole, and cerebellum (all *P* < 0.001) in patients. Besides, WM volume loss was also found in the OFC, GR, and insula (all *P* < 0.001) in patients. All WM atrophy areas were connected to areas of GM volume loss spatially. Correlation analysis showed the olfactory scores were significantly positively correlated with the GM volume of the occipital cortex (*P* < 0.001, and *P*_FWE_ < 0.05), while no significant correlation was found between the Sniffin' Sticks test scores and the WM volume in patients.

**Conclusion:**

The reduction of GM and WM volume in olfactory-related regions was responsible for olfactory dysfunction in post-traumatic patients. The occipital cortex may play a compensation mechanism to maintain the residual olfactory function. To our knowledge, we report here for the first time on white matter volume alterations specifically in post-traumatic patients with anosmia.

## Introduction

Traumatic brain injury (TBI) is one of the main causes of olfactory disorder and is also a significant public health event recognized worldwide (1). Patients with post-traumatic olfactory dysfunction (PTOD) are frequently presented with other clinical symptoms involving cognitive, emotional, and motor impairments that can seriously affect their quality of life (2). The frequency of olfactory dysfunction after traumatic brain injury was 13.7 and 8.2% had anosmia, accounting for 39% of patients seeking consultation from smell and taste clinics (3, 4). Unfortunately, there are limited treatment options and the prognosis of olfactory disorders secondary to head trauma is worse than other causes including sinonasal and post-viral olfactory dysfunction (5). It is critical to fully understand the comprehensive pathophysiological mechanisms, especially in the peripheral and brain central cortex. According to the previous studies, several mechanisms had been proposed to describe the possible pathophysiology of PTOD including sinonasal tract disruption, direct shearing or stretching of olfactory nerve fibers at the cribriform plate, and focal contusion or hemorrhage within the olfactory cortices (6, 7) and patients with PTOD performed as impaired odor thresholds, odor discrimination, and odor identification (8).

Voxel-based morphometry (VBM) is a useful method to analyze the morphological changes in the brain as measured by whole-brain MRI data and allows for the quantification of regional gray matter (GM) and white matter (WM) volumes of the cortex. Previous studies have demonstrated significant alteration of GM volume of the olfactory-related regions in patients with olfactory dysfunction due to different causes including idiopathic factors (9–11), chronic sinusitis (12), and neurodegenerative diseases (13) or heterogeneous etiologies (14). However, little study has focused on the cerebral GM changes in patients with PTOD, even though the VBM methods had been utilized for detecting the GM alterations for patients with TBI. Furthermore, those studies are always focused on GM's alterations, ignoring whether there are differences in WM of those brain regions. To our knowledge, there were several studies exploring the alteration of both the GM volume and WM volume in the olfactory regions among patients with mixed etiologies of olfactory loss (15, 16), but there were none for patients with post-traumatic anosmia. Recent studies used VBM methods to explore the brain MRI image of patients with TBI and showed reduced GM and WM volume in patients with traumatic brain injury (17, 18). Whether there were altered GM volumes and their association with olfactory function in patients with PTOD is largely unknown.

In the present study, the first aim of this study was to examine brain GM and WM volumes in patients with post-traumatic anosmia as compared with healthy controls. The second aim was to determine whether GM volumes in olfactory related regions was correlated with the patient's residual olfactory function.

## Methods and Materials

### Subjects

A total of 40 participants with right-handed took part in the study, including 18 of them being healthy controls (10 men and eight women; mean age = 34.78 years, SD = 13.69 years), and 22 patients who were diagnosed with PTOD (nine men and 13 women; mean age = 40.91 years, SD = 11.06 years) according to the Position paper on olfactory dysfunction. All the participants were collected for demographical and clinical characteristics, including age, gender, GM, and WM volume of the whole brain and olfactory function scores tested by sniffin' sticks (see later). The participants were screened for without psychiatric disorders, nasal sinus disease or drug use associated with olfactory dysfunction. In addition, all the post-traumatic patients with anosmia had no impaired olfactory function before head injury. All the participants underwent a complete physical and nasal endoscopy performed by an experienced rhinologist, and scores on their Simple Mental State Examination (MMSE) were normal. This study was approved by the Ethics Committee at Beijing Anzhen Hospital (Beijing, China; Approval No. 2019015X) and complied with the Declaration of Helsinki for Medical Research involving Human Subjects. All the participants provided written informed consent prior to the participation.

### Olfactory Function Test

For each participant, psychophysical testing of olfactory function was performed by using Sniffin' Sticks tests (Burghart, Gmbh, Wedel, Germany) and olfactory threshold (OT), olfactory discrimination (OD), olfactory identification (OI), and the overall composite scores (TDI; composite score of threshold, discrimination, and identification) were evaluated. Sniffin' Sticks have been assessed in healthy Chinese adults and various patients with olfactory dysfunction in our previous studies (19, 20). Sniffin' Sticks is suitable for application in the Chinese population to differentiate normosmia from hyposmia and anosmia (21). Standard administration was performed according to the manufacturer's instructions. For the testing, felt-tip pens containing various odors were presented to the participants. The pen's tip was placed ~2 cm in front of both nostrils for bilateral stimulation. The test comprised three parts: OT, OD, and OI test. The overall results were combined and reported as TDI scores ranging from 1 to 48, with higher scores indicating superior olfactory performance. A TDI scores 31, 16–30, and <15 indicated normosmia, hyposmia, and anosmia, respectively (22).

### Structural Brain Image Acquisition and Voxel-Based Morphometry

The whole-brain MRI was performed on a 3.0T (General Electric Company, America) GE scanner with a 16-channel phased-array head coil. For each subject, axial T1-weighted image (in total 188 slices) were acquired using an IR-prepped 3D fast gradient echo T1-weighted sequence (BRAVO) sequence with the following parameter: voxel size: 0.47 × 0.47 × 1.0 mm; repetition time: 7.5 ms; echo time: 2.8 ms; flip angle: 15°, field-of-view: 256 mm. The BRAVO sequence could provide isotropic voxels resulting in the higher spatial resolution images, which present crisper anatomic details to identify the lesions compared with the conventional scanning sequence (23). Furthermore, the sequence is optimized during the TI (time of inversion) process to provide excellent gray-white matter contrast, enabling gray-white matter segmentation and local volume measurements (24), which makes the results more accurate.

Voxel-based morphometry of T1-weighted images was performed using the CAT12 software (http://dbm.neuro.uni-jena.de/vbm/) implemented in SPM12 (Welcome Centre of Imaging Neuroscience, Institute of Neurology, UCL, London, UK; http://www.fil.ion.ucl.ac.uk/spm) and MATLAB (version 2013a, The MathWorks, Natick, MA, USA). After checking the data quality, the T1 images were first segmented into GM, WM, and cerebrospinal fluid (CSF). The GM and WM images were spatially normalized to a template in Montreal Neurological Institute (MNI) space using the high-dimensional Diffeomorphic Anatomical Registration Through Exponentiated Lie Algebra (DARTEL). Then, the image and preprocessing quality in the catreports were rechecked. Finally, the normalized GM and WM images were smoothed with a Gaussian kernel (full width at half maximum 8 mm). Automated data quality checks were performed as per the CAT12 toolbox. The volumes of GM, WM, and CSF of each participant were summed up to the total intracranial volume (TIV).

Voxel-wise GM and WM difference between the study groups (healthy controls and post-traumatic patients with anosmia) were examined using independent-sample *t*-tests, with gender, age and TIV as variables. A whole-brain threshold of *p* < 0.001 uncorrected was considered as statistically significant (25), and was used in combination with a non-stationary threshold to balance the risks of Type-I and Type-II errors (26). The non-stationary cluster extent threshold was computed within the xjview (http://alivelearn.net/xjview). Significant activations were located with the AAL toolbox (27). Correlations between the GM and WM volume and olfactory scores of patients and healthy controls were analyzed using the multiple regression design in SPM12 with age, gender, and TIV as covariates. The purpose of using covariables was to eliminate the adverse effects of these parameters on the results. Correlation analyses were considered significant at a whole-brain threshold of *P* < 0.001 uncorrected and peak wise *P* < 0.05 family wise error (FWE) corrected with a non-stationary cluster extent threshold.

### Statistical Analysis

All the statistical analyses of demographical, clinical characteristics, the GM and WM volumes of the whole brain and TIV between the two groups were performed with SPSS software version 24.0 (IBM Corporation, New York, NY, USA). Before statistical analysis, all the data will be tested by the Kolmogorov–Smirnov Test to verify whether it conforms to the normal distribution. Continuous variables were presented as mean ± SD, median (with range or interquartile range) or percentage according to the data distribution. Chi-square tests were used to compare frequencies for the categorical variables. Two-tailed values of *P* < 0.05 were considered statistically significant.

## Result

### Participants' Demographical and Clinical Results

The demographical and clinical characteristics of the enrolled participants are shown in Table 1. No significant differences were found between the post-traumatic patients with anosmia and healthy controls with respect to age, gender, right-handedness, smoker, GMV, WMV, and TIV (*P* > 0.05 for all or Chi-square test *P* > 0.05). Post-traumatic patients with anosmia had a significantly lower odor threshold (OT) score, odor discrimination (OD) score, odor identification (OI), and threshold-discrimination-identification (TDI) score (*p* < 0.05). The TDI score of all the patients was <15 and post-traumatic anosmia was diagnosed.

**Table 1:** Demographical and clinical characteristics of the cohort.

**Characteristic**	**Patient with post-traumatic anosmia** **(*n* = 22)**	**Healthy controls** **(*n* = 18)**	***P*** **-value**
Age (years), mean ± SD^a^	40.91 ± 11.06	34.78 ± 13.69	0.125
Male, n (%)^b^	9(40.9%)	10(55.6%)	0.356
TWM, mean ± SD^a^ (mm^3^)	527.17 ± 61.53	537.54 ± 58.41	0.591
TGM, mean ± SD^a^ (mm^3^)	624.84 ± 64.38	655.27 ± 61.38	0.137
TIV, mean ± SD^a^ (mm^3^)	1,532.52 ± 151.18	1,571.59 ± 153.74	0.425
OT score, median (IQR)^c^	1(1, 1.13)	6.38(5.38, 7.50)	<0.001
OD score, median (IQR)^c^	5(3.75, 6.5)	14(13, 15)	<0.001
OI score, median (IQR)^c^	4(4, 5.25)	15(14, 16)	<0.001
TDI score, mean ± SD^a^	11.67 ± 3.35	35.28 ± 1.81	<0.001

*SD, standard deviation; IQR, interquartile range; OT, odor threshold; OD, odor discrimination; OI, odor identification; TDI, threshold, identification and discrimination; TIV, total intracranial volume; TGM, total gray matter volume; TWM, total white matter volume*.

a *Analysis was performed by independent sample t-test*.

b *Analysis was performed by the χ^2^ test*.

c *Analysis was performed by Mann–Whitney U-test. Two-tailed values of P < 0.05 were considered statistically significant*.

### GM Volume

**Figure 1:**
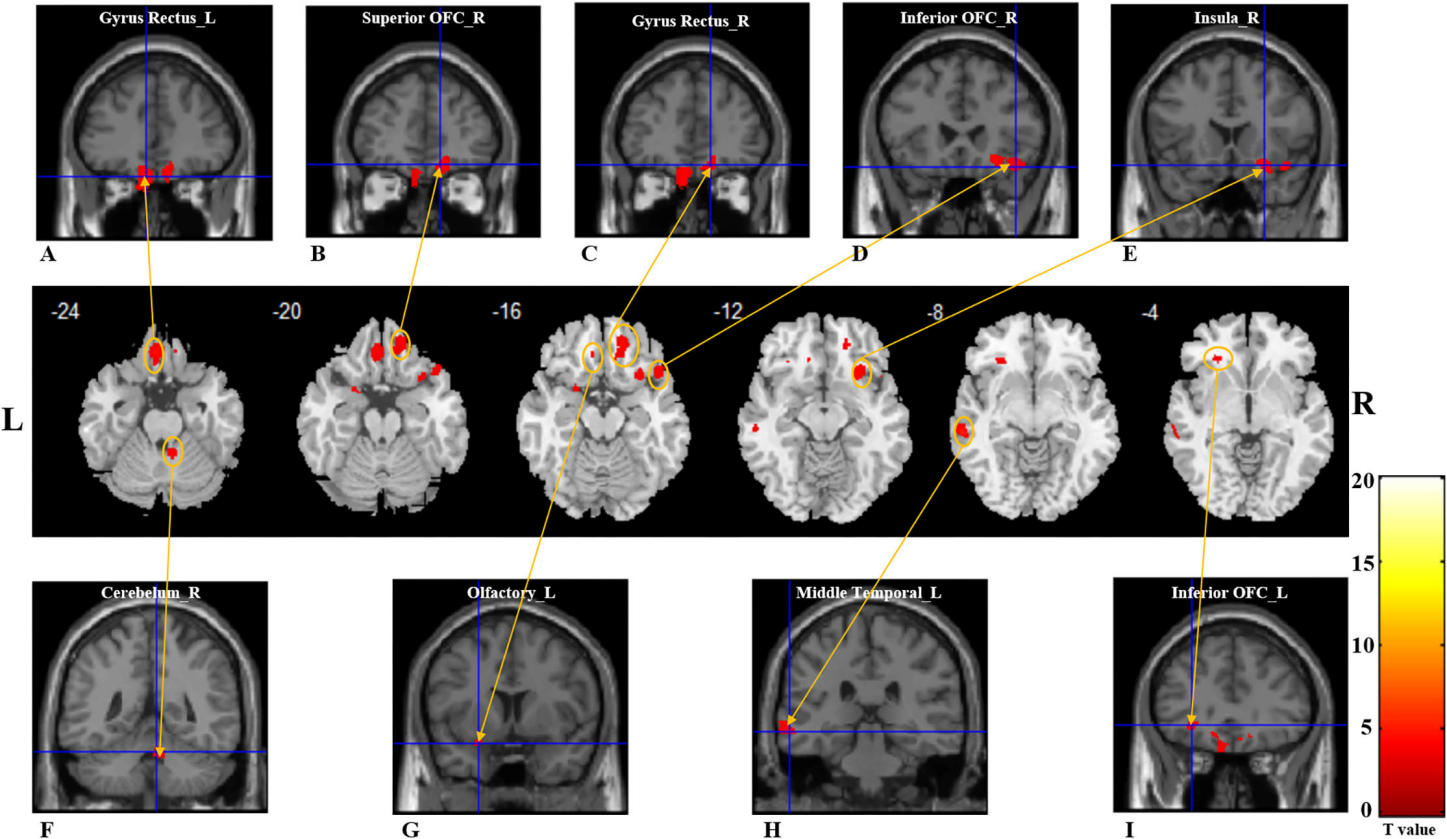
Gray matter reductions in post-traumatic patients with anosima compared with healthy controls. The VBM result is thresholded at *P* < 0.001. Whole-brain analyses with *P*_uncorrected_ < 0.001, and a cluster-extent threshold applied: *k* ≥ 36. **(A–I)** With red color represents the decreased GM volume regions in the patient group; **(A)** left gyrus rectus; **(B)** right superior OFC; **(C)** right gyrus rectus; **(D)** right inferior OFC; **(E)** right insula; **(F)** right cerebellum; **(G)** left olfactory cortex; **(H)** left middle OFC; **(I)** left inferior OFC. GM, gray matter; L, Left; R, Right; OFC, Orbitofrontal cortex.

Compared to healthy controls, patients with PTOD with anosmia had significantly reduced GM volume in the left rectus extending to the left superior OFC, the right rectus and superior OFC extending to the right middle OFC, the left middle temporal lobe, the right inferior extending to right superior temporal pole, the left inferior OFC, the cerebellum, the left primary olfactory cortex, insula, parahippocampal, and superior temporal pole (cluster extent threshold *P* < 0.001, cluster size ≥ 39 voxels). Besides, patients with anosmia had increased GM volume in the left Paracentral Lobule and precentral, and the superior parietal compared with the healthy controls (Table 2; Figure 1).

**Table 2:** Brain regions demonstrated gray matter volume alterations in post-traumatic patients with anosmia.

	**Region (AAL)**	**Side**	**Cluster size (voxels)**	***T*** **-value**	**Shared cluster^※^**	**MNI coordinates (mm)**		
						***x***	***y***	***z***
Patients < Control	Gyrus rectus	L	605	4.5118	40.0%	−6	38	−24
	Superior OFC	L			7.2%			
	Superior OFC	R	386	4.3154	44.8%	14	45	−17
	Gyrus rectus	R			36.3%			
	Middle OFC	R			3.5%			
	Middle temporal	L	217	5.5441	75.9%	−60	−30	−9
	Inferior OFC	R	150	4.0935	60.0%	44	20	−17
	Superior temporal pole	R			31.3%	44	20	−17
	Cerebellum	R	136	4.1326	99.3%	6	−46	−26
	Inferior OFC	L	76	4.4449	88.2%	−30	31.5	−6
	Olfactory cortex	L	39	3.7039	11.8%	−24	7.5	−18
	Insula	L			7.4%			
	Parahippocampal gyrus	L			9.6%			
	Superior temporal pole	L			5.9%			
Patients > Control	Paracentral lobule	L	235	4.5801	89.0%	−15	−21	75
	Precentral	L			62.5%			

*Whole-brain analyses with P_uncorrected_ < 0.001, and a cluster-extent threshold applied: k ≥ 39 for the Main effect T-test*.

*L, left; R, right; OFC, Orbitofrontal cortex; AAL, automated anatomical labeling; MNI, Montreal Neurological Institute*.

※ *When more than one region in the cluster, regions are reported with > 1% shared clusters*.

### WM Volume

**Figure 2:**
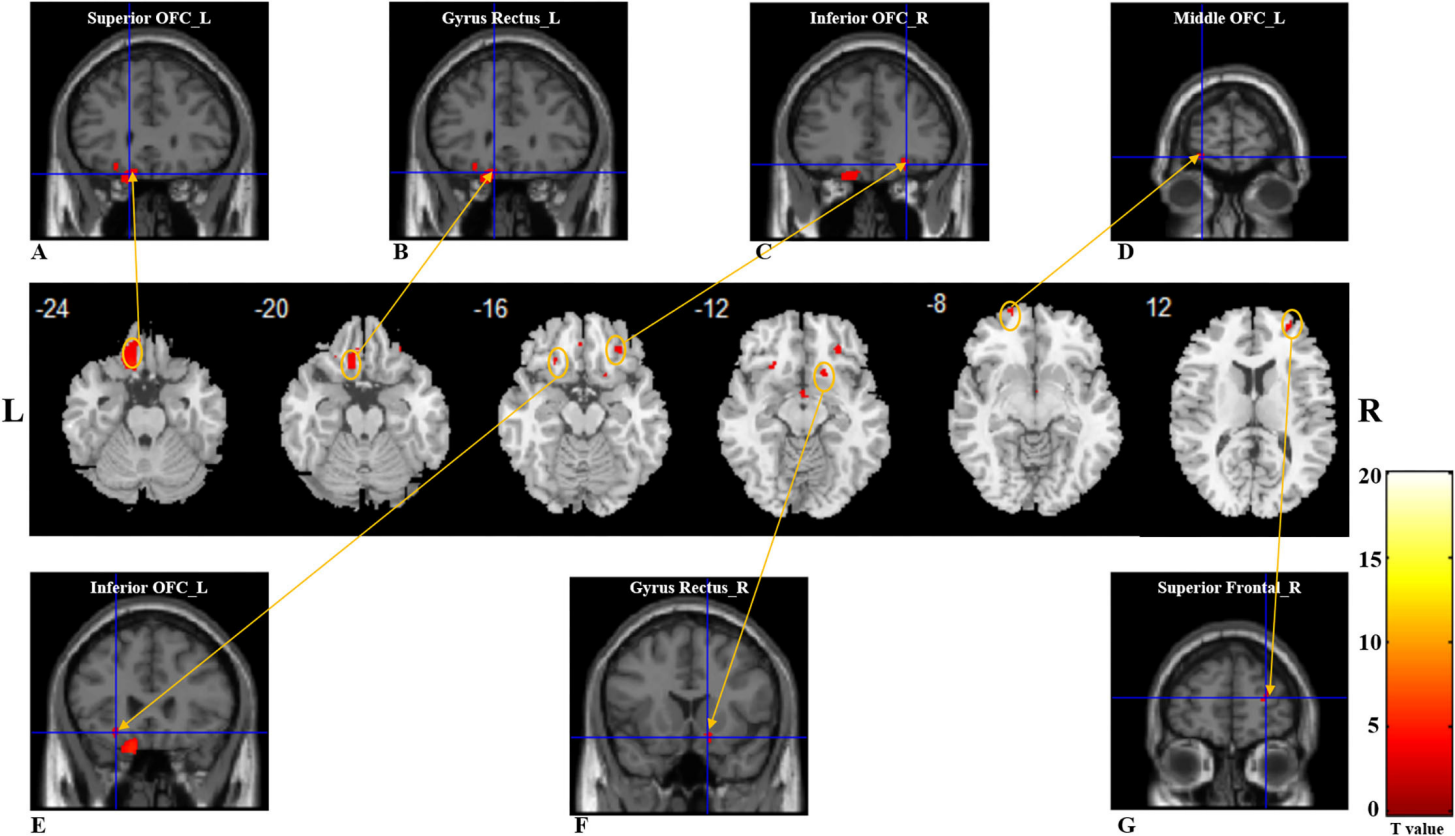
White matter reductions in post-traumatic patients anosima compared with healthy controls. The VBM result is thresholded at *P* < 0.001. Whole-brain analyses with *P*_uncorrected_ < 0.001, and a cluster-extent threshold applied: *k* ≥ 36 for the main effect *t*-test. **(A–G)** with red color represents the decreased WM volume regions in the patient group. **(A)** left superior OFC; **(B)** left gyrus rectus; **(C)** right inferior OFC; **(D)** left middle OFC; **(E)** left inferior OFC; **(F)** right gyrus rectus; **(G)** right superior frontal. VBM, voxel-based morphometry; L, Left; R, Right; OFC, Orbitofrontal cortex.

Compared with healthy controls, patients with PTOD with anosmia had significantly reduced WM volume in the left superior OFC and rectus extending to inferior OFC, right superior frontal lobe, right superior, middle and inferior OFC, left superior, middle, and inferior OFC extending to the insula, and right rectus extending to caudate (cluster extent threshold *P* < 0.001, cluster size ≥ 36 voxels). However, there was no increased WM volume in patients with anosmia (Table 3; Figure 2).

**Table 3:** Brain regions demonstrated white matter volume alterations in post-traumatic patients with anosmia.

	**Region (AAL)**	**Side**	**Cluster size (voxels)**	***T*** **-value**	**Shared clusters^※^**	**MNI coordinates (mm)**		
						***x***	***y***	***z***
Patients < controls	Superior OFC	L	705	6.0445	42%	−14	23	−29
	Gyrus rectus	L			15%			
	Inferior OFC	L			4%			
	Superior OFC	R	94	4.3154	77%	29	56	12
	Middle OFC	R	92	4.1986	52%	29	36	−15
	Inferior OFC	R			37%			
	Superior OFC	R			8%			
	Inferior OFC	L	66	3.7372	80%	−26	26	−14
	Insula	L			14%			
	Superior OFC	L	58	4.4324	71%	−21	66	−9
	Middle OFC	L			14%			
	Gyrus rectus	R	36	3.648	89%	15	17	−14
	Caudate	R			11%			

*Whole-brain analyses with P_uncorrected_ < 0.001, and a cluster-extent threshold applied: k ≥ 36 for the Main effect T-test*.

*L, left; R, right; OFC, Orbitofrontal cortex; AAL, automated anatomical labeling; MNI, Montreal Neurological Institute*.

※ *When more than one region in the cluster, regions are reported with > 1% shared clusters*.

### Correlation Results

For patients with post-traumatic anosmia, the OD and the TDI scores were positively correlated with GM volume with the right superior occipital cortex, while no correlation was found between OT, OI scores, and GM volume. For healthy controls, only the OI score was positively correlated with GM volume with the right superior frontal cortex (Table 4). No correlation was found between the Sniffin' Sticks test scores and the WM volume in patients or healthy controls.

**Table 4:** Correlation between olfactory scores and regional gray matter volume.

	**Olfactory scores**	**Correlation**	**Regions (AAL)**	**Side**	**Cluster size (voxels)**	***T*** **-value**	**Shared clusters^※^**	**MNI coordinates (mm)**		
								***x***	***y***	***z***
Patients	OD	Positive	Superior occipital cortex	R	355	5.2711	92%	23	−77	18
	TDI	Positive	Superior occipital cortex	R	396	6.6088	85%	20	−86	24
Controls	OI	Positive	Superior frontal cortex	R	312	6.1334	58%	26	56	17
			Middle frontal cortex	R			39%	26	56	17

*Whole-brain analyses with P_uncorrected_ < 0.001, and peak wise P_FWE_ < 0.05 corrected*.

*L, Left; R, Right; OD, odor discrimination; OI, odor identification; TDI, threshold, identification and discrimination; AAL, automated anatomical labeling; MNI, Montreal Neurological Institute*.

※ *When more than one region in the cluster, regions are reported with > 1% shared clusters*.

## Discussion

**Figure 3:**
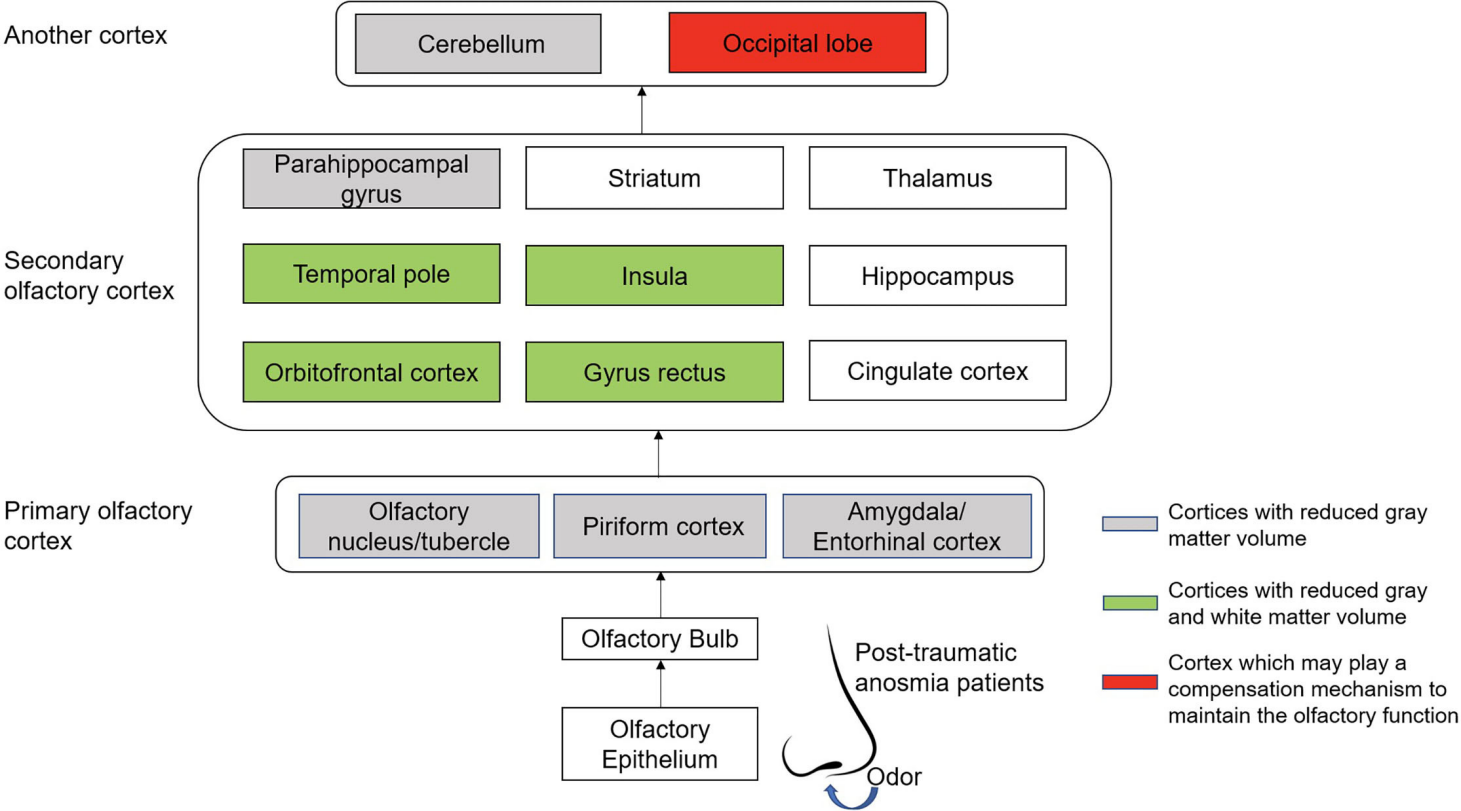
The connections and patterns of gray and white matter volume alterations in patients with post-traumatic anosmia. The olfactory-related regions' connections in post-traumatic patients with anosmia are mainly from the nasal cavity olfactory mucosa through nerve fiber to the occipital lobe and cerebellum, including the primary olfactory cortex and the secondary olfactory cortex. The cortices with decreased gray matter volume because of the trauma were mainly located in the primary olfactory cortex and the cerebellum. In the secondary olfactory cortex, areas such as the orbitofrontal cortex, the gyrus rectus, the temporal pole, and the insula were found to be associated with decreased gray matter volumes and white matter volumes. In addition, the occipital lobe may play a role in maintain the residual olfactory function of post-traumatic patients when the conventional olfactory cortices were damaged.

To our knowledge, this is the first study to focus on the gray and white matter volume alteration in post-traumatic patients with anosmia as determined by VBM using SPM12. We found that patients with post-traumatic anosmia presented with decreased gray matter volume in the OFC, bilateral GR, olfactory cortex, insula, parahippocampal gyrus, temporal pole, and cerebellum (Figure 3). In addition, the WM matter volume got decreased in bilateral OFC and GR extending to the insula and caudate. All the white matter atrophy areas were spatially connected to areas of gray matter volume loss except the parahippocampal, temporal pole, and cerebellum. However, the patients got increased GM volume from the left paracentral to the precentral lobule. The correlation analysis showed that the right superior occipital cortex was positively correlated with OD and TDI scores, respectively.

In the present study, we found that OFC and GR's GM volume decreased, accompanied by decreased WM volume. The OFC and GR play essential roles in the process of olfactory and were considered the secondary olfactory cortex, mainly responsible for odor perception (28). Besides, studies suggested that the OFC and GR also connected with odor thresholds and the level of olfactory expertise (29, 30). OFC accepted the projection of the primary olfactory cortex (28), and also received projections from the thalamic nucleus, which involved olfactory and flavor perception (31). Head trauma can directly damage the olfactory bulb and the frontal lobe and is often associated with impaired olfactory recognition, which had been confirmed by the previous study (32). Meanwhile, our study showed the OI scores of the healthy group was positively correlated with the volume of the right frontal cortex, indicating that the frontal cortex plays an important role in odor identification. In our previous studies, it was also found that the glucose metabolism was decreased in OFC and GR regions of post-traumatic patients with anosmia (33). However, the GM reduction of OFC was not only found in patients with post-traumatic anosmia, but also in patients with olfactory loss secondary to other causes (10, 12). Therefore, OFC volume change can be substantial evidence of brain morphologic changes in patients with olfactory dysfunction. Consistent with our findings, reduced WM volume of OFC was found in patients with anosmia with mixed etiologies (15, 16). Our results suggested that the cause of PTOD is not only the damage of neurons in those brain regions, but also the damage or atrophy of nerve fibers.

Insula was another region that got reduced both GM volume and WM volume in our study. The insula was considered to be the secondary olfactory cortex and received fibrous projections from the primary olfactory cortex, especially from the anterior piriform cortex and amygdala (28). From previous studies, the insula has been identified to be involved in odor perception (34, 35), odor discrimination (36), and odor hedonic (37). Consistent with previous studies (15), our results also found that the volume of WM extending from OFC to insula was reduced, indicating the atrophy of fiber bundles between the high-level cortices involved in olfactory discrimination and recognition. Besides, it has been proved that olfactory hallucination was the main symptom of right posterior insula cerebral ischemia (38). More important, insula is responsible for integrations of olfaction with taste and trigeminal integrations (39). And direct stimulation of insula could cause the sensory of gustatory and olfactory (40). These evidences may support the fact that patients with post-traumatic anosmia always company with taste dysfunction.

Our results show a reduction in the olfactory cortex, extending to the parahippocampal gyrus and temporal pole. However, those regions were not associated with changes in the WM volume. The olfactory cortex, receiving direct input from olfactory bulb, is considered as the primary olfactory area which comprises the olfactory tubercle, the anterior olfactory nucleus, the piriform cortex, the amygdaloid cortex, and the lateral entorhinal cortex (41), and play roles on olfactory perception *via* attentional mechanism to designate the identical flow of air through the nose (42). The reduced GM volume in the primary olfactory cortex among patients with post-traumatic anosmia suggests decreased peripheral olfactory inputs. The temporal pole is a highly interconnected area that is well-known recipient of projections from amygdala, OFC, piriform cortex, and insula, playing roles in odor emotional responses (43), and odor recognition (44), indicating the importance of this area for retention of namable odors. Like the temporal pole, parahippocampal is closely related to olfactory memory (45). In patients with PTOD, reduced GM volume of the temporal pole and parahippocampal gyrus may lead to the occurrence of distorted olfactory processing (parosmia) after injury (46). In addition, patients with post-traumatic anosmia have been shown to exhibit GM reduction in the cerebellum when compared with healthy controls, which was consistent with previous studies (47, 48). The cerebellum is also involved in olfaction, especially with activation in relation to sniffing and alterations of the respiratory pattern (49). Therefore, post-traumatic patients with anosmia with breathing pattern changes are possible due to the GM volume reduction in the cerebellum.

A most significant finding in the present study was that a positive association was observed between the OD and DTI scores with the GM volume in the occipital cortex among patients with post-traumatic anosmia. The occipital is not a regular olfactory-related brain region identified by the researchers. A study by Chen and colleagues demonstrated that patients with congenital Parkinson and olfactory dysfunction showed decreased GM volume of occipital, consistent with our result (13). Meanwhile, among volunteers with normal sense of smell, extensive activation related to the occipital cortex was found after odor stimulation (50). An earlier study found that circuits associated with occipital were involved in processing hedonic and edibility judgments of odors (51). A comparative study of patients with congenital blindness and healthy volunteers found that in the absence of vision, olfactory stimulation would preferentially enter the occipital area, so occipital is believed to be related to olfactory processing (52). Unfortunately, previous studies did not clarify how the occipital area specifically exerted its olfactory effect. From the aspect of anatomy, the occipital got extensive fiber connections with temporal and frontal lobe (53) which contained the olfactory secondary centers responsible for odor memory and perception. Therefore, based on our current results, we inferred that occipital played a role in odor discrimination in the process of olfaction, which required future research to verify.

The present study had several limitations. First, our study was cross-sectional and sample of patients (*n* = 22) and controls (*n* = 18) was not very large, further longitudinal or larger sample size studies were warranted to consolidate findings of this study. Second, our study did not show the alterations of the olfactory bulb's volume, which was considered a significant important factor affecting olfactory function (48, 54, 55). Further studies we will use tools such as VBM to accurately measure olfactory bulb volume to observe the influence of head trauma on olfactory bulb volume and olfactory function. Third, the olfactory-related cortex has the characteristics of plasticity (56), and the olfactory function of traumatic brain injury patients often recover spontaneously, and most of them occur 6 months to 1 year after injury (57). Although the disease duration of the patients we recruited in our study was <1 year, it was not clear that their brain structure was remodeled during the spontaneous recovery.

## Conclusions

Our study demonstrated alterations of the GM and WM volumes of olfactory related regions in patients with post-traumatic anosmia. Atrophy of GM volume and WM volume in the OFC, GR, and insula cortex was highly associated with olfactory dysfunction in post-traumatic patients. The occipital cortex may play a compensation mechanism to maintain the residual olfactory function. To our knowledge, we report here for the first time on white matter volume alterations in post-traumatic patients with anosmia.

## Data Availability Statement

The raw data supporting the conclusions of this article will be made available by the authors, without undue reservation.

## Ethics Statement

The studies involving human participants were reviewed and approved by Ethics Committee at Beijing Anzhen Hospital (Beijing, China; Approval No. 2019015X). The patients/participants provided their written informed consent to participate in this study.

## Author Contributions

XG analyzed the date and drafted the manuscript. BS and ZS contributed to the methodology and interpretation of data. LX contributed to the vital instruments and medicines. YW and DW designed the study, revised the manuscript, supervised the study, and obtained the funding. All authors have approved the submitted version, and agree both to be personally accountable for our contributions and to ensure that questions related to the accuracy or integrity of any part of the work, even ones in which we are not personally involved, are appropriately investigated, resolved, and the resolution documented in the literature.

## Funding

DW was supported by grants from the Beijing Hospitals Authority Youth Program (QML20190617), Beijing Science and Technology Nova Program (Z201100006820086), Natural Science Foundation of China (82000954), and Beijing Hospitals Authority Clinical Medicine Development of Special Funding (XMLX202136). YW was supported by grants from the Beijing Hospitals Authority's Mission Plan (SML20190601), Beijing Scholars Program (No. 051), and National Key R&D Program of China (No. 2019YFE0116000).

## Conflict of Interest

The authors declare that the research was conducted in the absence of any commercial or financial relationships that could be construed as a potential conflict of interest.

## Publisher's Note

All claims expressed in this article are solely those of the authors and do not necessarily represent those of their affiliated organizations, or those of the publisher, the editors and the reviewers. Any product that may be evaluated in this article, or claim that may be made by its manufacturer, is not guaranteed or endorsed by the publisher.
